# Loss of tolerance to gut immunity protein, glycoprotein 2 (GP2) is associated with progressive disease course in primary sclerosing cholangitis

**DOI:** 10.1038/s41598-017-18622-1

**Published:** 2018-01-10

**Authors:** Tamas Tornai, David Tornai, Nora Sipeki, Istvan Tornai, Rayan Alsulaimani, Kai Fechner, Dirk Roggenbuck, Gary L. Norman, Gabor Veres, Gabriella Par, Alajos Par, Ferenc Szalay, Peter Laszlo Lakatos, Peter Antal-Szalmas, Maria Papp

**Affiliations:** 1 Department of Internal Medicine, Division of Gastroenterology, Faculty of Medicine, University of Debrecen, Debrecen, Hungary; 2 Department of Laboratory Medicine, Faculty of Medicine, University of Debrecen, Debrecen, Hungary; 3 Institute of Experimental Immunology, Euroimmun AG, Lübeck, Germany; 4 Institute of Biotechnology, Faculty Environment and Natural Sciences, Brandenburg University of Technology Cottbus-Senftenberg, Senftenberg, Germany; 5 GA Generic Assays GmbH, Dahlewitz, Germany; 6 Inova Diagnostics, Inc., San Diego, CA USA; 7 1st Department of Pediatrics, Semmelweis University, Budapest, Hungary; 8 1st Department of Medicine, University of Pecs, Pecs, Hungary; 9 1st Department of Medicine, Semmelweis University, Budapest, Hungary

## Abstract

Glycoprotein 2[GP2] is a specific target of pancreatic autoantibodies[PAbs] in Crohn’s disease(CD) and is involved in gut innate immunity processes. Our aim was to evaluate the prevalence and prognostic potential of PAbs in primary sclerosing cholangitis(PSC). Sixty-five PSC patients were tested for PAbs by indirect immunofluorescence and compared with healthy (n = 100) and chronic liver disease controls(CLD, n = 488). Additionally, a panel of anti-microbial antibodies and secretory (s)IgA levels were measured, as markers of bacterial translocation and immune dysregulation. PAbs were more frequent in PSC(46.2%) compared to controls(healthy:0% and CLD:4.5%), [P < 0.001, for each]. Occurrence of anti-GP2 antibody was 30.8% (20/65) and was exclusively of IgA isotype. Anti-GP2 IgA positive patients had higher sIgA levels (P = 0.021). With flow-cytometry, 68.4% (13/19) of anti-GP2 IgA antibodies were bound with secretory component, suggesting an active retro-transportation of anti-GP2 from the gut lumen to the mucosa. Anti-GP2 IgA was associated with shorter transplant-free survival [P_LogRank_ < 0.01] during the prospective follow-up (median, IQR: 87 [9–99] months) and remained an independent predictor after adjusting for Mayo risk score(HR: 4.69 [1.05–21.04], P = 0.043). These results highlight the significance of gut-liver interactions in PSC. Anti-GP2 IgA might be a valuable tool for risk stratification in PSC and considered as a potential therapeutic target.

## Introduction

Primary sclerosing cholangitis (PSC) is a chronic cholestatic liver disease characterized by persistent, progressive biliary inflammation and fibrosis. Clinical manifestations and progression of the disease are heterogeneous and lack reliable biomarkers. Consequently, patients with PSC cannot be stratified appropriately^1^. Until now only a clinical score, the Mayo risk score, has been proved useful in risk assessment of the disease^2^. While the etiology of the disease is poorly understood, a dysregulated interplay of key genetic and immunologic pathways with the microbiome appears to contribute to the development of PSC and associated inflammatory bowel diseases (IBD)^3^. A more detailed exploration of derangement in gut-liver axis might lead to assist the development of disease specific therapy. There is an unmet need for effective medical treatments for PSC, and to date, the only curative therapy is liver transplantation reserved for those with end-stage liver disease^4^.

Enhanced formation of autoantibodies against acinar cells of the exocrine pancreas have long been described in Crohn’s disease (CD) and considered one of the various serological hallmarks of the disease^5^. Recently, the target antigens of pancreatic autoantibodies (PAbs) have been identified: the pancreatic major glycoprotein of the zymogen granule membrane (GP2, also referred to as MZGP2) and CUB and zona pellucida-like domain-containing protein 1 (CUZD1)^6,7^. Further investigations revealed that GP2 plays an important role in intestinal immune responses^8,9^. Previously, we demonstrated in a large cohort of patients with IBD^10^, that the presence of PAbs was associated with concomitant PSC and also with faster disease progression in patients with CD. Interestingly, anti-GP2 and anti-CUZD1 antibodies predicted different features of disease progression and notably, only IgA but not IgG antibodies were related to the development of complications. In addition, various other target specific auto- and anti-microbial IgA antibodies were associated with pathologic bacterial translocation in cirrhosis, and thus the development complications^11,12^. The gut-associated lymphoid tissue (GALT) plays a central role in the IgA antibody formation. Occurrence of serological antibodies directed against gut innate immune proteins or intestinal microorganisms could be a clue of an excessive mucosal immune response due to extended microbial challenge or dysregulation. Association between serological antibodies linked to enteric bacteria and the progressive disease course in PSC is a reasonable hypothesis^13,14^.

IgA is an important humoral component of intestinal mucosal immunity. During transportation of the molecule through the epithelial cell to the mucosal surface, a cleaved fragment of the polymeric IgA receptor (secretory component, SC) remains covalently attached to the dimeric IgA molecule and this complex is referred to as secretory IgA (sIgA). The molecule has a dual function. It plays a role in neutralizing microbial antigens and in preventing interaction with the intestinal epithelium, a mechanism called immune exclusion. Secretory IgA is also involved in the retro-transportation of antigens to GALT that has important immunological functions^15^. After retro-transportation of sIgA molecules, they reach the systemic circulation via the thoracic duct and thus can be detectable in sera. In healthy individuals, however, the percentage of SC containing IgA in terms of the total IgA pool is less than 1%.

The aims of this study were to investigate^1^: the prevalence and type of target specific PAbs in a mixed cohort of pediatric and adult PSC patients with and without IBD^2^; the association between PAbs and both clinical and laboratory characteristics of the disease^3^; whether the presence of PAbs is associated with a progressive disease course in PSC; and^4^ the possible mechanisms related to the formation of PSC-associated PAbs.

## Results

### Frequency of target-specific PAbs in PSC

Frequencies of IgA and IgG isotype PAbs in PSC and various control groups comprising healthy blood-donors and both IBD and CLD patients are summarized in Table 1. A total of 46.2% (30/65) of PSC patients were positive for either one of the two target-specific PAbs (anti-CUZD1 and anti-GP2), with a significant difference compared to healthy controls (0.0%, P < 0.001) and all disease control groups (CD: 26.8%, P < 0.001 and UC: 7.6%, P < 0.001 or aLC: 6.7%, chrHCV: 4.2% and PBC: 4.9%, P < 0.001 for all CLD). Distribution of the two types of PAbs in PSC was equal, 30.8% (20/65) for each. One-third of the positive cases (10/30) showed double positivity (anti-CUZD1 and anti-GP2). Anti-GP2 antibody positivity was exclusively IgA isotype, while anti-CUZD1 antibodies were of both IgA and IgG isotypes.

**Table 1:** Target-specific anti-pancreatic antibodies (PAbs) in patients with primary sclerosing cholangitis (PSC) and various healthy and diseases control groups.

	**PSC (N = 65)**		Inflammatory bowel disease (N = 427)						Healthy control			Chronic liver diseases (N = 488)								
			CD (N = 257)			UC (N = 170)			HC (N = 100)			aLC (N = 267)			chr HCV (N = 119)			PBC (N = 102)		
	**%**	**n**	%	n	p-value	%	n	p-value	%	n	p-value	%	n	p-value	%	n	p-value	%	n	p-value
Anti-GP2 IgA	**30**.**8**	**20**	6.2	16	<0.001	0	0	<0.001	0	0	<0.001	4.9	13	<0.001	4.2	5	<0.001	3.9	4	<0.001
Anti-GP2 IgG	**0**	**0**	7	18	0.030	0	0	x	0	0	x	0	0	x	0	0	x	0	0	x
Anti-CUZD1 IgA	**18**.**5**	**12**	12.1	31	0.175	4.7	8	0.001	0	0	<0.001	1.9	5	<0.001	0	0	<0.001	1	1	<0.001
Anti-CUZD1 IgG	**20**	**13**	16.3	42	0.484	5.9	10	0.001	0	0	<0.001	0.4	1	<0.001	0	0	<0.001	0	0	<0.001
Anti-CUZD1 (IgA or IgG)	**30**.**8**	**20**	21	54	0.095	7.6	13	<0.001	0	0	<0.001	2.2	6	<0.001	0	0	<0.001	1	1	<0.001
Anti-CUZD1 and Anti-GP2	**15**.**4**	**10**	3.5	9	<0.001	0	0	<0.001	0	0	<0.001	0	0	<0.001	0	0	<0.001	0	0	<0.001
Anti-CUZD1 or Anti-GP2	**46**.**2**	**30**	26.8	69	<0.001	7.6	13	<0.001	0	0	<0.001	6.7	18	<0.001	4.2	5	<0.001	4.9	5	<0.001

CD: Crohn’s disease, UC: ulcerative colitis, HC: healthy controls, aLC: alcoholic liver cirrhosis, chr HCV: chronic hepatitis C virus, PBC: primary biliary cholangiopathy, PSC: primary sclerosing cholangitis.

PAbs: pancreatic autoantibodies, GP2: glycoprotein 2, CUZD1: CUB and zona pellucida-like domain 1.

p-values pertain to comparisons between PSC and the given control group, x: non applicable.

### Association between target-specific PAbs and clinical or laboratory characteristics of PSC

We analyzed clinical and laboratory characteristics of PSC patients according to their PAb status and summarized these results in Table 2.

**Table 2:** Associations between different target-specific anti-pancreatic antibodies (PAbs) and clinical or laboratory characteristics of primary sclerosing cholangitis (PSC) at enrolment.

	Anti-GP2 IgA		p-value	Anti-CUZD1 IgA		p-value	Anti-CUZD1 IgG		p-value
	Negative (n = 45)	Positive (n = 20)		Negative (n = 53)	Positive (n = 12)		Negative (n = 52)	Positive (n = 13)	
Male gender	68.9% (31)	75% (15)	0.617	71.7% (38)	66.7% (8)	0.729	71.2% (37)	69.2% (9)	0.892
Presence of cirrhosis	**11.1% (5)**	**35% (7)**	**0.022**	15.1% (8)	33.3% (4)	0.141	21.2% (11)	7.7% (1)	0.263
Presence of IBD	77.6% (38)	70% (14)	0.638	73.6% (39)	75% (9)	0.92	69.2% (36)	92.3% (12)	0.09
Ulcerative Colitis	46.9% (23)	60% (12)	0.138	43.4% (23)	66.7% (8)	0.234	44.2% (23)	61.5% (8)	0.234
Crohn’s Disease	30.6% (15)	10% (2)		30.2% (16)	8.3% (1)		25% (13)	30.8% (4)	
Age at diagnosis (yr)	23 (17–37)	23.5 (18–33)	0.764	25 (18–37)	18 (15–27)	0.098	25 (17–37)	19 (16–27)	0.118
Disease duration (yr)	**7 (3**–**10)**	**4 (2**–**7)**	**0.027**	**6 (4**–**10)**	**2 (1**–**5)**	**0.002**	6 (3–10)	3.5 (2–6.5)	0.183
Albumin (g/L)	**45 (42**–**47)**	**40 (39**–**43)**	**0.001**	44 (40–47)	40 (37–43)	0.116	**44 (40**–**47)**	**40 (37**–**41)**	**0.019**
Bilirubin (μmol/L)	15 (11–20)	17 (11–34)	0.383	15 (10–21)	17 (12–37)	0.188	15 (11–22)	20 (15–24)	0.355
AST (U/L)	**32 (25**–**43)**	**69 (50**–**96)**	**<0.001**	37 (28–61)	51 (36–80)	0.168	42 (28–70)	41 (36–56)	0.672
ALT (U/L)	**38 (21**–**64)**	**90 (56**–**165)**	**0.002**	47 (24–100)	85 (40–165)	0.207	50 (27–104)	64 (40–100)	0.670
GGT(U/L)	**97 (44**–**208)**	**298 (141**–**499)**	**0.001**	147 (63–310)	193 (60–478)	0.430	160 (82–332)	142 (53–305)	0.758
ALP (U/L)	**420 (246**–**595)**	**898 (637**–**1532)**	**<0.001**	**516 (326**–**688)**	**902 (260**–**1800)**	**0.030**	552 (367–765)	901 (260–1068)	0.653
Mayo risk score	**−0.859 (−1.447**–**0.131)**	**−0.072 (−0.6**–**0.658)**	**0.010**	−0.6 (−1.378–0.174)	−0.321 (−0.834–0.292)	0.369	−0.579 (−1.286–0.203)	−0.564 (−0.775–0.073)	0.636

Variables are summarized as: percentage, (n) or median (25–75 percentile values).

ALP: alkaline phosphatase, ALT: alanine transaminase, AST: aspartate transaminase, CUZD1: CUB and zona pellucida-like domains 1, GGT: gamma glutamyl transferase, GP2: glycoprotein 2, PAbs: pancreatic autoantibodies, IQR: inter-quartile range

Significant associations are indicated in bold.

PAbs demonstrated no association with gender and younger age at diagnosis. Several laboratory and clinical parameters, indicating more severe disease were significantly higher in the presence of anti-GP2 IgA antibody. Patients with anti-GP2 IgA antibodies had a significantly shorter disease duration (4 [2–7] vs. 7 [3–10] years, P = 0.009), and increased Mayo risk score. All liver enzymes were significantly elevated, while albumin level was decreased in anti-GP2 IgA positive cases compared to anti-GP2 negative ones. There was an elevated occurrence of cirrhosis in anti-GP2 IgA-positive patients (35% vs. 11.1%, P = 0.022). CUZD1 IgA-positivity was associated with shorter disease duration and higher ALP, while CUZD1 IgG-positivity with lower albumin level. There was no difference in Mayo risk score according to anti-CUZD1 antibody status. The presence and subtype of concomitant IBD were similar according to PAb status.

### Significance of target-specific PAbs in the risk of progressive disease course in PSC

Seven patients underwent prior orthotopic liver transplantation (OLTx) during the follow-up period. In all cases the indication was the development of end-stage liver disease. Six patients died due to liver-related complications, three out of six deaths occurred after OLTx, therefore the composite end-point (OLTx and/or liver-related death) occurred in a total of 10 patients. One patient died due to acute myocardial infarction, this case was censored at time of event. Median follow-up from inclusion was 2632 [IQR: 286–3022] days. Development of colon cancer occurred in 2, while biliary tract cancer in 1 patient. Nine of involved patients had at least one episode of cholangitis.

We analyzed the association of clinical variables and the different PAbs with poor disease outcome. In Kaplan-Meier analysis, the median time to OLTx and/or liver-related death was 490 days (IQR: 49–1033). Mayo risk score and the presence of cirrhosis (P_Log-rank_ < 0.001 and =0.007, respectively), but not gender (P_Log-rank_ = 0.441), age at onset (P_Log-rank_ = 0.884), disease location (P_Log-rank_ = 0.722) or concomitant IBD (P_Log-rank_ = 0.432) were significantly associated with faster disease progression (Supplementary Figure 1 and Supplementary Table 1).

**Figure 1:**
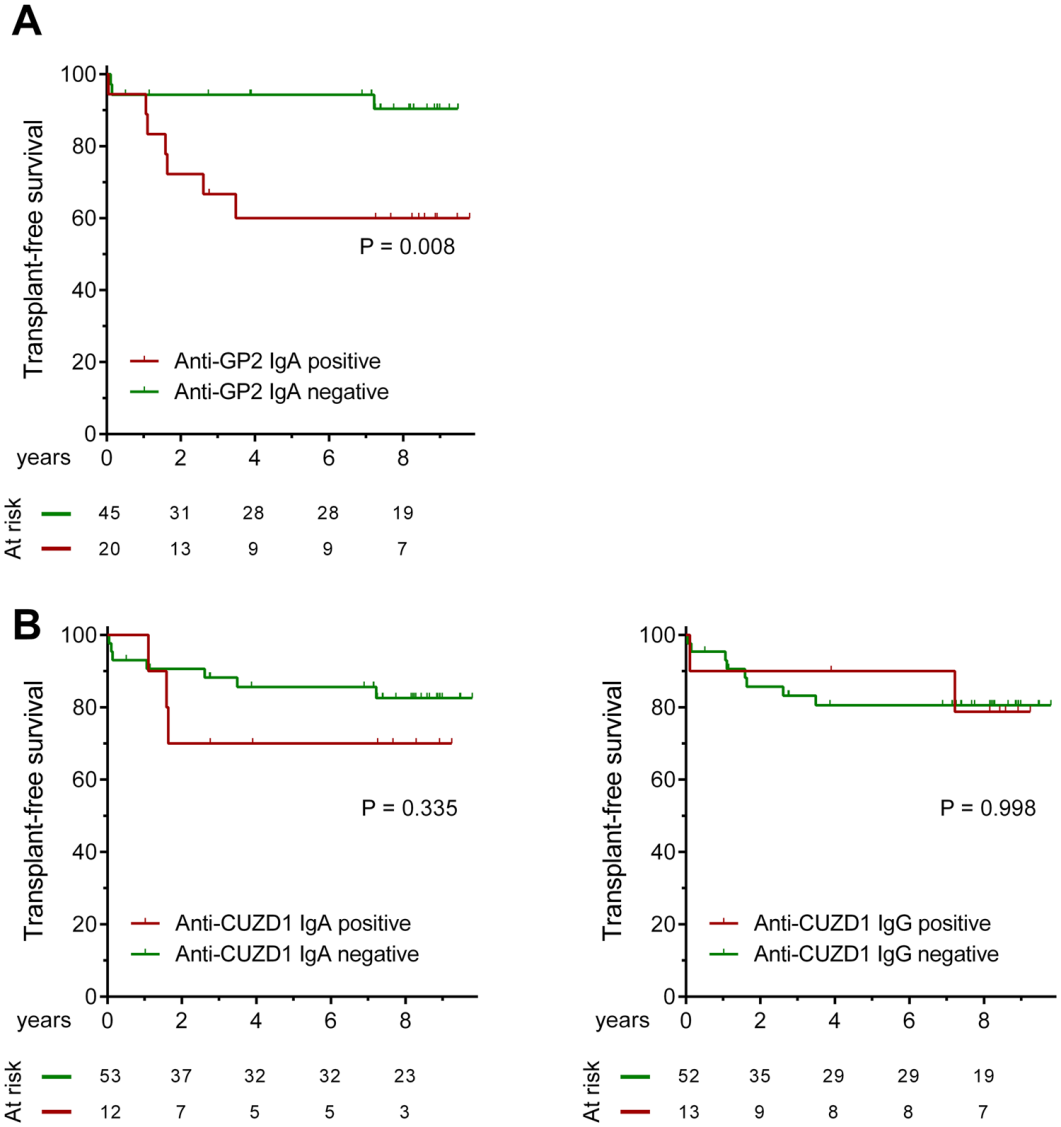
Progressive disease course in primary sclerosing cholangitis (PSC) according to the presence of different target-specific anti-pancreatic antibodies. Patients positive for anti-GP2 IgA have higher cumulative probability of disease progression defined by need for OLTx and/ or death compared to those negative for anti-GP2 IgA antibody. (**A**) Neither anti-CUZD1 IgA and nor anti-CUZD1 IgG were associated with the progressive disease course (**B**) GP2: glycoprotein 2, CUZD1: CUB and zona pellucida-like domain-containing protein 1.

Positivity for IgA isotype anti-GP2 antibody (P_Log-rank_ = 0.008), but not for IgA or IgG isotype anti-CUZD1 antibodies (P_Log-rank_ = 0.335 and 0.998, respectively) predicted OLTx and/or liver-related death, see Fig. 1. Accordingly, univariate Cox regression analysis (Supplementary Table 1) revealed anti-GP2 IgA-positivity as a predictor for poor disease outcome (HR: 5.15 [1.33–19.97], P = 0.018), that remained an independent predictor after adjusting for Mayo risk score in multivariate Cox-regression analysis (HR: 4.69 [1.05–21.04], P = 0.043). A similar tendency was found when we adjusted for the presence of cirrhosis, instead of Mayo risk score (HR: 3.74 [0.9–15.55], P = 0.07). In sensitivity analysis, positivity for anti-GP2 IgA was associated with poor outcome in the subgroup of patients with adult onset PSC (P_Log-rank_ = 0.034) and concomitant IBD (P_Log-rank_ < 0.001), but not in the subgroup of patients with pediatric onset PSC (P_Log-rank_ = 0.098) and no concomitant IBD (P_Log-rank_ = 0.666).

### Significance of classic serological markers in the risk of progressive disease course in PSC

We also determined the frequency of classic serological markers in our PSC cohort. A total of 83.1% (54/65) and 28.1% (18/64) of PSC patients were positive for IgA/IgG atypical P-ANCA and ASCA and significantly different from the healthy controls (4%, P < 0.001 and 16%, P = 0.04). Regarding Ig isotypes, atypical P-ANCA was primarily IgG, while ASCA was equally IgG or IgA: 83.1% vs. 40% and 18.8% vs. 21.9%, respectively. Furthermore, 18.8% and 9.4% of the patients were positive for IgA type anti-OMP Plus^TM^ and EndoCab antibody, respectively. However, the frequency of these latter serological antibodies did not differ from those of healthy controls (20% and 4.7%).

**Figure 2:**
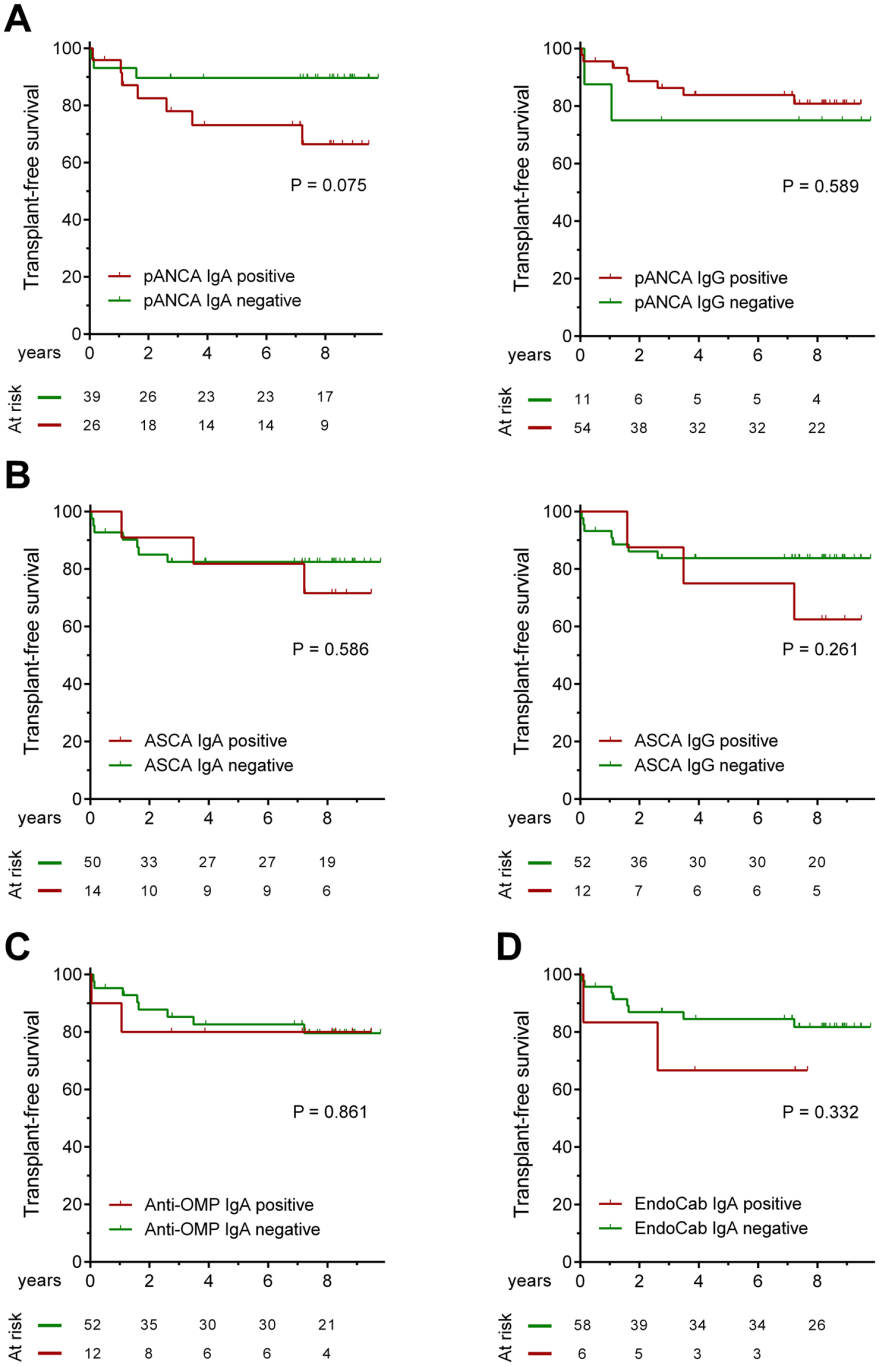
Progressive disease course in primary sclerosing cholangitis (PSC) according to the presence of various classic serological markers. Neither IgA and nor IgG atypical P-ANCA (**A**) ASCA (**B**) anti-OMP (**C**) and EndoCab antibodies (**D**) were associated with the progression of disease. ASCA: anti-*Saccharomyces cerevisiae* antibodies, P-ANCA: perinuclear anti-neutrophil cytoplasmic antibody, EndoCab: endotoxin-core antibody.

Association of these antibodies with poor disease outcome was also assessed in Kaplan-Meier analysis. Neither IgG nor IgA isotypes of these serological antibodies were associated with the risk of the progressive disease course nor were corresponding IgA isotypes, see Fig. 2.

### Anti-GP2 IgA antibody status is associated with increased secretory IgA concentration

Serum levels of total sIgA were significantly higher in patients with PSC compared to healthy controls (median [IQR], 96 [73–180] vs. 30 [21–42] μg/ml, P < 0.001). Furthermore, anti-GP2 IgA positive cases had higher sIgA levels compared to anti-GP2 IgA negative ones (149 [86–246] vs. 89 [71–118] μg/ml, P = 0.021). No difference in serum sIgA levels were observed in case of other serological antibodies (Table 3).

**Table 3:** Serum level of total secretory (s)IgA (mg/L) according to the different serologic antibody statuses.

	Serum level of secretory IgA (median (IQR), mg/L)		p-value
	negative	positive	
ANTIBODIES			
Anti-GP2 IgA	**89 (71**–**118)**	**149 (86**–**246)**	**0.021**
Anti-CUZD1 IgA	93 (71–160)	149 (85–293)	0.108
Anti-CUZD1 IgG	95 (74–187)	97 (60–126)	0.376
Atypical P-ANCA IgA	100 (71–183)	89 (74–143)	0.810
Atypical P-ANCA IgG	100 (71–115)	95 (74–185)	0.7
ASCA IgA	98 (74–185)	91 (71–143)	0.782
ASCA IgG	96 (73–181)	99 (72–217)	0.783
Anti-OMP Plus IgA	95 (71–176)	111 (77–196)	0.449
EndoCab IgA	94 (73–183)	130 (71–156)	0.836

ASCA: anti-*Saccharomyces cerevisae* antibody, CUZD1: CUB and zona pellucida-like domains 1, EndoCab: endotoxin core antibody, GP2: glycoprotein 2, P-ANCA: perinuclear anti-neutrophil cytoplasmic antibody, IQR: inter-quartile range

Significant associations are indicated in bold.

### Characterization of GP2 antibodies

**Figure 3:**
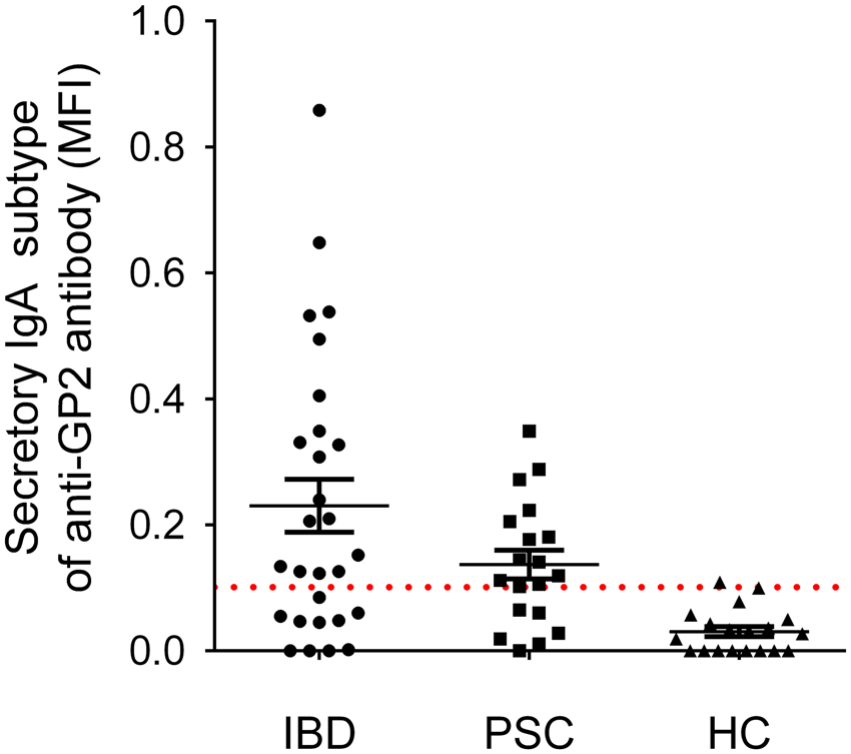
Presence of secretory component on GP2 IgA antibodies. Cut-off positivity (0.101) was defined by the mean + 2 SD median MFI value of the negative control group (healthy subjects).

Finally, we characterized anti-GP2 IgA antibodies in patients with PSC and Crohn’s disease in a flow-cytometry subtyping assay. The presence of secretory component (SC) on GP2 IgA antibodies was 68.4% in PSC (13/19) similar to the frequency (75%,9/12) observed in patients with CD without PSC, see Fig. 3.

## Discussion

Previously, we observed high frequency of PSC in anti-GP2-positive CD patients^10^. We, as well as others^16^ also reported associations of novel target-specific PAbs (anti-GP2 and anti-CUZD1) with various aspects of progressive disease course in CD patients. Interestingly only IgA, but not IgG isotype antibodies were related to the development of complications in that prospective, large-scale study^10^. Therefore, we aimed to investigate the prevalence and clinical importance of different target-specific PAbs in a prospective PSC cohort with respect to different immunoglobulin isotypes.

Simultaneously with a research group from Germany, we demonstrated enhanced IgA type anti-GP2 antibody formation in PSC^17,18^. We used the same cell-based IIF test with an identical cut-off value. As shown elsewhere, IIF test appears to detect anti-GP2 IgA in patients with PSC better in contrast to ELISA^10^. The IIF test used in this study detects the interaction of IgA with glycosylphosphatidylinositol (GPI)-anchored GP2 on the surface of HEK239 cells^6^. This might provide a unique epitope structure of GP2 which could be different from one of GP2 adsorbed to ELISA solid phases. A careful comparison with the findings of *Jendrek et al*. published as a full paper recently^19^ revealed however, some differences. In our PSC patient cohort, occurrence of anti-GP2 IgA was lower (30.8% vs. 48.7%), while that of anti-CUZD1 antibodies was higher (IgA: 18.5% vs. 9.4% and IgG: 20% vs. 6.3%). The reason for these differences is not fully understood, but is most probably attributed to the composition of the patient populations. First, the Mayo risk score reflecting disease severity was higher in their cohort. Second, the occurrence of biliary tract cancer was 12.3%, while none of our patients had CCA at baseline. Third, in that study anti-GP2 IgA positivity was not exclusively associated with PSC, but rather with the presence of large bile duct diseases, irrespectively of their malignant or benign character based on findings in their disease control groups. Finally, in our study, we used an extensive CLD group with various etiology as disease controls in contrast to the study of *Jendrek et al*. Neither patients with small bile duct disease (primary biliary cholangitis, PBC n = 102), nor patients with alcoholic liver cirrhosis showed increased PAb frequency. This later finding is particularly intriguing, because in cirrhosis we found high frequency of various serological antibodies, mainly of IgA isotype, such as ASCA (38.5%) or ANCA (52.2%)^11,12^ that are also frequent in PSC.

Exploring patient characteristics at enrolment, the presence of anti-GP2 IgA-positivity was associated with a more severe disease phenotype. Consistently, anti-GP2 IgA predicted faster disease progression during follow-up, even after adjusting for the Mayo risk score or the presence of cirrhosis. In our study, both liver-related death and OLTx were considered as equal endpoints, since they represent the development of end-stage liver disease as the result of the progressive fibrosis. In the parallel study of *Jendrek et al*., anti-GP2 IgA-positivity also identified a subgroup of patients with high mortality. Notably, poor survival was primarily attributed to cholangiocarcinoma (CCA) and not to the development of end-stage liver disease in their cohort. In our cohort, only 1 patient (1.5%) developed biliary tract cancer during follow-up, therefore evaluation of PAbs regarding the development of CCA was not possible. This CCA rate is lower than the reported 8–13.2% in other studies^20,21^, but equals with those ones from Israel (2.1%) and the Netherlands with corresponding follow-up times in recent reports^22,23^. Supporting clinical evidence for the anti-GP2 IgA - fibrosis linkage in PSC might be the observation that anti-GP2 IgA was more prevalent in CD patients with stricturing disease in previous reports^10,16,24^.

Formation of anti-GP2 antibody represents the breakdown of tolerance towards the GP2 protein. Glycoprotein 2, an innate immunity protein released from the exocrine pancreas into the intestinal lumen, is also expressed on the apical membrane surface of M-cells. There GP2 can interact with FimH-positive bacteria bearing type-1 fimbriated pyli. GP2-mediated transcytosis is necessary for the initiation of antigen-specific mucosal immune responses against this type of bacterial antigen^25^. Interestingly we showed, that there is an enhanced immunological response to a wide range of other enteric bacterial antigens in PSC as well. These were as follows: phosphopeptidomannan (ASCA), different endotoxins (EndoCab) and multiple Gram-negative and Gram-positive proteins (OMP). In autoimmune liver disorders, atypical P-ANCA was reported to be directed against human β-tubulin isotype-5 (TTB-5), a protein which shares an extraordinary high structural homology with the microbial cell division protein (FtsZ). FtsZ is present in almost all bacteria in the intestinal microflora. Therefore, occurrence of P-ANCA has been considered a sign of immunological response to enteric bacteria^13^. However, in our study, serological response to these bacterial antigens was not associated with disease progression in PSC.

To explore the possible link between mucosal immunity and the development of severe phenotype in PSC, the serum levels of total sIgA were measured. A novel finding of our study is that in PSC, the total sIgA levels were significantly elevated, namely three-fold higher, compared to healthy controls. These results indicate an elevated retro-transport of sIgA from the gut mucosal surface in PSC. An additional two-fold elevation of total sIgA levels were found in anti-GP2 IgA-positive cases that was not seen in the presence of other serological anti-microbial antibodies or ANCA. These results suggest that retro-transport is specifically further enhanced in patients with anti-GP2 IgA-positivity. Flow-cytometry based characterisation of anti-GP2 IgA-positive samples revealed that SC was present on these molecules in up to 68.4% of cases. This high percentage of SC strongly indicates that after secretion to the gut lumen, anti-GP2 IgA antibodies are also retro-transported across the mucosal epithelium. Retro-transported sIgA molecules, however, are not “lonely” particles, but rather exist in partnership with their antigens. Since antigen-coupled sIgA molecules are those that have high affinity to their epithelial receptors^26^, anti-GP2 IgA antibodies grab GP2-coated FimH-positive bacteria as companions. In this way enhanced retro-transport of anti-GP2 IgA might increase microbial overload in the mucosal compartment and perpetuate antigen-induced signalling. Memory T lymphocytes primed in the inflamed gut and homing to the biliary tract via aberrantly expressed adhesion molecules plays a fundamental role in the extension of gut inflammation to the biliary tract^27^. Interestingly, FimH has been recently identified as a ligand of Toll-like receptor (TLR)-4^28^. Sustained TLR4 activation leads to enhanced fibrosis through TGF-beta signaling^29^. At the same time, FimH elicits an immune response with enhanced type I Interferon production that has been linked to disease amplification in autoimmunity^30^. These mechanisms could serve an explanation of how the breakdown of tolerance towards GP2 in the gut is associated with the development of enhanced fibrosis, and thus disease progression in the liver. The GP2—FimH axis deserves further exploration in the pathogenesis of PSC and might also be a highly intriguing issue from the therapeutic point of view. GP2 has a high structural and functional homology with uromodulin (Tamm-Horsfall protein in the urinary tract)^9^. Recombinant vaccine against the adhesion protein of FimH is already under development in recurrent urinary tract infections. Furthermore mannose-derived FimH antagonists, also hold promise as a novel treatment for UTIs and Crohn’s disease^31–33^.

In summary, the presence of target-specific PAbs is associated with PSC and its more severe phenotype, but not with other CLDs including cholestatic liver disease with small bile duct involvement. The findings of our prospective referral cohort study indicate that anti-GP2 IgA may be a useful additional serological tool in the stratification of PSC patients and is associated with the progressive disease course. In addition, occurrence of IgA type anti-GP2 antibody may serve as an additional clue towards the significance of gut-liver interactions in the disease course of PSC.

## Methods

### Patient population

We performed an observational cohort study among adult and pediatric PSC patients recruited in Hungarian referral hepatology centers (Hungarian Autoimmune Liver Disease Study Group). This study population is equal with the patient cohort published previously^34^, with exception that 4 patients were excluded due to prior liver transplantation in this study. In total 65 well-characterized PSC patients with a complete clinical follow-up (adult: 55 [male/female: 39/16], median age at presentation: 28 years [IQR: 19–37], median disease duration: 6 years [2–10] and children: 10 [male/female: 7/3], median age at presentation: 10 years [IQR: 6–11], disease duration: 5 [1.5–7.5]) were included between January, 2006 and December, 2007. Clinical characteristics of patients at enrolment are presented in Table 4.

**Table 4:** Clinical and laboratory characteristics of patients with primary sclerosing cholangitis (PSC).

	Patients with PSC (n = 65)	
**Median (IQR)**		
Age at diagnosis (years)	23	(17–37)
Disease duration (years)	6	(2–8)
Albumin (g/L)	43	(40–46)
Bilirubin (µmol/L)	15	(11–23)
ALT (U/L)	52	(32–100)
AST (U/L)	42	(28–69)
GGT (U/L)	154	(63–320)
ALP (U/L)	566	(326–822)
INR	1.0	(1.0–1.2)
Platelet (10^9^/L)	257	(187–321)
Mayo risk score	−0.564	(−1.142–0.174)
**% (n)**		
Children	15.4%	(10)
Male	70.8%	(46)
Cirrhosis	20.3%	(14)
IBD	73.9%	(48)
Crohn’s Disease	35.4%	(17)
Ulcerative Colitis	64.6%	(35)
Overlap syndrome	13.8%	(9)
Small duct PSC	7.7%	(5)

ALP: alkaline phosphatase, ALT: alanine transaminase, AST: aspartate transaminase, GGT: gamma glutamyl transferase, INR: international normalized ratio, IBD: inflammatory bowel disease, IQR: inter-quartile range

The entire cohort comprised 69 patients but those with prior liver transplantation (n =  4) were excluded from the study.

Diagnosis of PSC was based on clinical, biochemical, serological and cholangiographic (magnetic resonance or endoscopic imaging) features or, when indicated, on histological findings^35^. Patients with any concomitant malignant disease were excluded. Blood samples and detailed description of clinical phenotypes were obtained at inclusion. Clinical data were determined by thorough review of patients’ medical records, which had been collected in a uniform format. Medical records that documented disease phenotype (age at onset of disease, duration [time elapsed from diagnosis until study inclusion], type of PSC – large duct or small duct), presence and type of concomitant IBD, presence of overlap syndrome, presence of cirrhosis and portal hypertension related complications (e.g. ascites, encephalopathy, oesophageal varices or variceal bleeding), prior orthotopic liver transplantation (OLTx), co-morbidities and medication (e.g. ursodeoxycholic acid, steroid, immunosuppressive and/or biological therapy) at inclusion were retrospectively analyzed for the period prior to the prospective follow-up. At enrolment, revised Mayo risk score was calculated^2^ and biochemical analyses were performed using standard routine laboratory protocols for tests including platelet count, creatinine, total bilirubin, albumin, international normalized ratio (INR) of prothrombin time, aspartate aminotransferase (AST), alanine aminotransferase (ALT), alkaline phosphatase (ALP), and γ-glutamyl-transferase (GGT).

### Phenotypical characterization of PSC patients during prospective follow-up

Phenotypical characterization of this patient cohort was performed previously and is provided similarly as the publication^34^. PSC patients were enrolled into a prospective follow-up study, where treating physicians registered laboratory data, imaging and endoscopic findings, medical treatment, date and type of complications (cirrhosis, colorectal cancer, biliary tract cancer: cholangiocarcinoma [CCA] gallbladder cancer [GBC] or cholangitis) during regular outpatient follow-up visits and inpatient stays. In Hungary, a follow-up visit is usually scheduled for every 6 months at a specialized hepatology center (the actual interval varies between 3–6 months). Collected data were transferred and stored in a database for analysis. On December 1^st^ 2015, all patients’ charts and data were reviewed and updated for the data points mentioned above. Adverse outcome was defined as need for OLTx and/or liver-related death (composite end-point). Follow-up for a particular patient was terminated if adverse outcome occurred or there was no further record available. One case with a non-liver related death (myocardial infarction) was censored at the time of event.

### Control groups

Healthy controls and two disease control groups (patients with inflammatory bowel diseases [IBD] and chronic liver diseases [CLD]) were included.

The healthy control group consisted of 100 age-matched individuals (male/female: 45/55, age: 30 years [21–40]) selected from consecutive blood donors in Debrecen. Control subjects did not have any known gastrointestinal or liver diseases.

The inflammatory bowel disease control group consisted of a previously reported patient cohort (CD: 257, male/female: 108/148, age: 25 [19–33] years and ulcerative colitis [UC]: 170, male/female: 78/92, age: 34 [23–44] years)^10^.

The chronic liver disease control group consisted of patients with primary biliary cholangitis without cirrhosis (PBC, n = 102, male/female: 4/98, age: 59 [52–66] years), chronic hepatitis C virus (chrHCV, n = 119, male/female: 50/69, age: 55 [47–65] years) and alcoholic liver cirrhosis (aLC, n = 267, male/female: 147/120, age: 58 [51–66] years). The diagnosis of PBC was based on biochemical evidence of cholestasis, serum anti-mitochondrial antibodies (AMA) and/or PBC-specific AMA-M2 positivity, compatible histology, and the exclusion of extrahepatic cholestasis^36^. The diagnosis of chronic HCV was based on positive HCV ribonucleic acid, elevated liver function tests (2xULN for more than 6 months) and compatible liver biopsy, if available. The diagnosis of cirrhosis was based on clinical, biochemical, imaging, and, when available, histological data.

### Serological Analysis

Blood samples were obtained at enrolment from each patient and were frozen at −70 °C until testing. All serological assays were performed in a blinded fashion without prior knowledge of the patient’s clinical information.

### Detection of target specific serological antibodies

#### Autoantibodies

IgA and IgG type anti-GP2 and anti-CUZD1 antibodies as well as atypical perinuclear anti-neutrophil cytoplasmic antibodies (P-ANCA) were detected in sera using commercially available cell-based IIFT according to the test instructions [CIBD Mosaic, EUROIMMUN Medizinische Labordiagnostika AG, Lübeck, Germany]. A specific fluorescence at a dilution of 1:10 or higher was considered positive for anti-GP2 and anti-CUZD1, while 1:32 or higher for atypical P-ANCA as recommended by the manufacturer. Detailed description of the protocols has been reported previously^10,12^ and given in the Supporting Material.

#### Anti-microbial antibodies

*Anti-Saccharomyces cerevisiae (ASCA)* (IgA and IgG, US FDA-cleared) and *anti-OMP Plus* (IgA) (research use only) antibodies were detected by commercially available sandwich enzyme-linked immunosorbent assays (ELISA) employing a specially purified preparation of *Saccharomyces cerevisiae antigen and* a mixture of multiple bacterial proteins derived from two species of intestinal bacteria (one Gram-positive and one Gram-negative) respectively, with a cut-off positivity at 25 U for all (QUANTA Lite^®^, Inova Diagnostics, San Diego, CA) as recommended by the manufacturer. *Endotoxin core IgA antibody (EndoCAb)* directed against a mixture of incomplete endotoxins of 4 different species (*Pseudomonas aeruginosa*, *Salmonella typhimurium*, *Escherichia coli* and *Klebsiella aerogenes*) was detected by commercially available ELISA (Hycult Biotechnology, Uden, Netherlands). The cut-off positivity was 195 AU/mL, defined as values exceeding the 95^th^ percentile level of the healthy control group.

### Detection of serological antibody of mucosal origin

Secretory subtype of total IgA (sIgA) was detected by an in-house sandwich ELISA in PSC and healthy controls. Detailed description of this experiment is presented in the Supporting Material.

### Characterization of IgA type anti-GP2 antibodies

We used GP2-coated bead-based in-house flow cytometric immunoassay to determine the presence of secretory component (SC) on anti-GP2 IgA antibodies in sera. Detailed description of this experiment is presented in the Supporting Material.

The IgA subtype analysis was performed in anti-GP2 IgA-positive sera of PSC (n = 19) and Crohn’s disease patients without PSC (n = 14) by IIFT and was also verified by ELISA test (anti-GP2 IgA, GA Generic Assays, Dahlewitz/Berlin, Germany). Anti-GP2 IgA-negative sera of healthy subjects (n = 20) served as controls.

### Ethical considerations

The study protocol was approved by the Regional and Institutional Research Ethics Committee of University of Debrecen and the National Scientific and Research Ethics Committee (DEOEC-RKEB/IKEB 5306–9/2011, 3515–2011, 3885/2012/EKU [60/PI/2012], 3880/2012/EKU [59/PI/2012]). Each patient was informed of the nature of the study and gave informed consent in writing. All methods were performed in accordance with the relevant guidelines and regulations^2,6,35–37^.

### Data availability statement

The datasets generated and analyzed during the current study are available from the corresponding author on reasonable request.

### Statistical methods

Variables were tested for normality using Shapiro Wilk’s W test. Continuous variables were summarized as medians (interquartile range [IQR, lowest 25% - highest 25%]) and were compared with the Mann-Whitney U test. Categorical variables were compared with χ^2^ test or Fisher’s exact test, as appropriate. Kaplan–Meier analysis was used to determine association between serological antibodies and adverse disease outcome (OLTx and/or liver-related mortality). Differences in observed survival were assessed by the log-rank test. The association of categorical clinical variables or serological antibodies with adverse disease outcome during the follow-up was evaluated by univariate Cox-regression analysis. Multivariate analyses were performed to adjust for the Mayo risk score or presence of cirrhosis with forced entry method. Associations are given as hazard ratio [HR] with 95% confidence intervals [CI]. For statistical analysis and graphical presentation, the SPSS v.22.0 (SPSS, Chicago, IL), and GraphPad Prism 6 (San Diego, CA) programs were used. A two-sided probability value of < 0.05 was considered to be statistically significant.

## Electronic supplementary material

Supplementary Material
