# Supplementary material for: Loss of tolerance to gut immunity protein, glycoprotein 2 (GP2) is associated with progressive disease course in primary sclerosing cholangitis
Source: Sci Rep. 2018 Jan 10;8:399. doi: 10.1038/s41598-017-18622-1 (PMC5762861; doi:10.1038/s41598-017-18622-1)
Supplement: Supplementary file 1 — Supplementary Material [file 41598_2017_18622_MOESM1_ESM.pdf]

Loss of tolerance to gut immunity protein, glycoprotein 2 (GP2) is associated with progressive disease course in primary sclerosing cholangitis

Authors: Tamas Tornai, MD, PhD[1] David Tornai, MD[2], Nora Sipeki, MD, PhD[1], Istvan Tornai, MD, PhD[1], Rayan Alsulaimani[2], Kai Fechner, MEng[3], Dirk Roggenbuck, MD, PhD[4,5], Gary L. Norman, PhD[6], Gabor Veres, MD, DSc[7], Gabriella Par, MD, PhD[8], Alajos Par, MD, DsC[8], Ferenc Szalay, MD, DsC[9], Peter Laszlo Lakatos, MD, DsC[9], Peter Antal-Szalmas, MD, PhD[2], Maria Papp, MD, PhD[1]

Affiliations:

[1]Department of Internal Medicine, Division of Gastroenterology, Faculty of Medicine, University of Debrecen, Debrecen, Hungary

[2]Department of Laboratory Medicine, Faculty of Medicine, University of Debrecen, Debrecen, Hungary

[3]Institute of Experimental Immunology, Euroimmun AG, Lübeck, Germany

[4]Institute of Biotechnology, Faculty Environment and Natural Sciences, Brandenburg University of Technology Cottbus-Senftenberg, Senftenberg, Germany

[5]GA Generic Assays GmbH, Dahlewitz, Germany

[6]Inova Diagnostics, Inc., San Diego, CA

[7]1st Department of Pediatrics, Semmelweis University, Budapest, Hungary

[8]1st Department of Medicine, University of Pecs, Pecs, Hungary

[9]1st Department of Medicine, Semmelweis University, Budapest, Hungary

Corresponding author: Maria Papp, MD, PhD, Department of Internal Medicine, Division of Gastroenterology, Faculty of Medicine, University of Debrecen, Debrecen, Hungary, 98 Nagyerdei krt., H-4032 Debrecen, Hungary, Phone/Fax: 36-52-255-152, e-mail: papp.maria@med.unideb.hu

Detection of target specific anti-pancreatic antibodies (PAbs)

IgA and IgG type anti-GP2 and anti-CUZD1 antibodies, and also atypical P-ANCA were detected in sera using cell-based IIFT according to the test instructions [CIBD Mosaic, EUROIMMUN Medizinische Labordiagnostika AG, Lübeck, Germany]. The slides contained a biochip mosaic consisting of transfected HEK293 cells expressing CUZD1 or GP2 [1] separately and mock-transfected HEK293 control cells, and also ethanol- and formalin-fixed human granulocytes. The slides were incubated with 25 μL pre-diluted sera [1:10, 1:100, 1:1000 and 1:32, 1:320 and 1:3200] in phosphate-buffered saline (PBS) per incubation field for 30 minutes at room temperature. Unbounded sample was then washed away with PBS-Tween and the slide was immersed in PBS-Tween for 5 minutes. In the second step, fluorescein isothiocyanate–labeled goat anti-human IgG or IgA (EUROIMMUN) were used to visualize bound antibodies of the patients' sera. Incubation time was 30 minutes at room temperature followed by another washing step as above. Slides were embedded in PBS-buffered glycerol (approximately 10 μL per field). Evaluation was performed using a Eurostar Plus microscope with Bluelight LED [EUROIMMUN Medizinische Labordiagnostika AG]. Positive and negative controls were included in each run. A specific fluorescence at 1:10 or higher for anti-CUZD1 and anti-GP2 antibodies, and a dilution of 1:32 or higher was considered positive for atypical P-ANCA. The interpretation of ANCA pattern was based on the behavior of the specimens on ethanol- and formalin- fixed slides according to previously reported. [2]

Detection of secretory subtype of total IgA (sIgA)

Secretory component (SC) on the total IgA pool (sIgA) was detected by in-house developed double-antibody sandwich enzyme-linked immunosorbent assay (ELISA) systems. Microtiter plates (flat bottom, high binding capacity, Greiner Bio-One, Mosonmagyarovar, Hungary) were coated by overnight incubation at 4°C with 3 μg/mL polyclonal sheep anti-human sIgA (Antibodies-Online GmbH, Aachen, Germany) diluted in carbonate/bicarbonate buffer (pH 9.6) Thereafter, wells were blocked with 1% bovine serum albumin (BSA,

Sigma-Aldrich, Mo, USA) in PBS with 0.02% Tween-20. Duplicates of pre-diluted sera (1:128) were then incubated for 60 min at room temperature (RT) together with a serial dilution of purified sIgA of human colostrum (Athens Research & Technology, USA) with known concentrations as a reference. After washing five times, specific horseradish peroxidise (HRP) conjugated anti-human IgA (α-chain specific) antibody (Sigma-Aldrich, Mo, USA) diluted 1:5000 in PBS containing 1% BSA at pH 7.4 was added and incubated for another 60 min at RT. Color was developed with tetramethyl-benzidine dihydrochloride (TMB, Sigma–Aldrich, Schnelldorf, Germany), stopped with 2M H_2SO_4, and read immediately at 450 nm in a Labsystem Multiscan MS plate reader (Thermo Scientific, Budapest, Hungary). The calculation of results was performed using the Genesis software program with four parametric curve fitting. Within-run, coefficients of variation (CV) was 4.6%, while between-run CV was 15.2%. The limit of detection was 0.03 μg/mL.

Characterization of IgA type anti-GP2 antibodies

The IgA subtype analysis was performed in the case of anti-GP2 IgA-positive sera of patients with PSC (n=18) and Crohn's disease (n=12) that were verified by ELISA test earlier (anti-GP2 IgA, GA Generic Assays, Dahlewitz/Berlin, Germany). Anti-GP2 IgA-negative sera of healthy subjects (n=20) served as the controls.

We used a GP2-coated bead-based in-house flow cytometric immunoassay to subtype anti-GP2 IgA in sera, namely IgA1, IgA2 and sIgA. Commercially available polystyrene beads (1.4×10^7 beads; Polysciences Inc., Warrington, PA, USA) - after 3 washing steps - were coated daily with 60 μg GP2 protein (provided by Prof. Dirk Roggenbuck, GA Generic Assays, Dahlewitz/Berlin, Germany) in 0.1 M carbonate buffer (pH 9.5) at RT for 16 hours. After 2 washing steps, beads were further incubated in phosphate buffered saline (PBS) containing 1% bovine serum albumin (BSA, HyClone, Logan, UT, USA) to avoid unspecific binding. GP2-coated and blocked beads (50 μL) at a concentration of 5×10^6 particles/mL were incubated with 50 μL of pre-diluted sera (1:5 in PBS containing 0.1% BSA) of patients and healthy controls for 30 minutes, at 4°C. After incubation, beads were washed twice

with PBS containing 0.1% BSA and unlabelled monoclonal mouse anti-human IgA1 (30 µg/mL, Antibodies-Online, Aachen, Germany), IgA2 (30 µg/mL, RayBiotech, Norcross, GA, USA) or SC (100 µg/mL, HyTest, Turku, Finland) were added. Samples were incubated with these antibodies at 4°C for 30 minutes. Unbounded primary antibodies were eliminated by two washing steps. To augment the signal 100 µL of secondary phycoerithrein (PE)-labelled polyclonal goat anti-mouse antibodies (1 µg/mL; DAKO, Glostrup, Denmark) were used. Incubation was performed again at 4°C for 30 minutes, and then specimens were washed and centrifuged according to the standard procedure. After decanting, pellets were dissolved in 400 µL paraformaldehyde (1%) and measured by a flow cytometer (Cytomics FC500, Beckman Coulter, USA). Beads were gated based on their forward and sideward scatter properties and the median PE intensity (FL2) was calculated. Data were recorded and analyzed using Kaluza Analysis Software (Beckman Coulter). In order to correct for background signal, blank samples were prepared for each patient serum where the IgA1/IgA2/SC specific antibody were omitted, but the other labelling steps were exactly the same as in the described staining procedure. These background values were subtracted from median fluorescence intensities (MFIs) of the real samples.

As we intended to determine the total anti-GP2 IgA level for each samples as a sum of the IgA2 and IgA1 subtypes of the antibody, we needed to standardize the fluorescence signal measured by the anti-IgA1 and anti-IgA2 antibodies. Quantum™ Simply Cellular® (QSC) microspheres (Bangs Laboratories, Inc., Fishers, IN, USA) coated with known number of anti-mouse antibodies (3,448; 19,426; 82,751; 276,941, antigen binding capacity [ABC]) were used to capture our mouse anti-human IgA1 or IgA2 antibodies plus the goat-anti mouse PE-labeled conjugate in the same dilutions that we applied in the previously described settings. The beads were analyzed by flow cytometry. The MFI of the beads was plotted against the known ABC and these calibration curves were used to determine the number of anti-GP2 antibodies bound to the GP2-coated particles. These estimated ABC values for IgA1 as well as IgA2 were used for calculating the total IgA level of anti-GP2 antibody, by the sum of their ABC values.

Summation of anti-GP2 IgA1 and IgA2 antigen binding capacity (ABC) values (total anti-GP2 IgA) measured by the novel flow cytometric assay in serum sample of patients and controls were compared to the anti-GP2 IgA titers determined by the ELISA system. There was a strong correlation ($r=0.879$, $p<0.001$), between the results of two different methods. The mean+2SD value of the anti-GP2 IgA ELISA negative samples gave an ABC cut-off of 9.425 by the flow cytometric method. All the patients' samples positive for anti-GP2 IgA antibody in ELISA showed flow cytometry values above this cut-off level corresponding to a total concordance between the two assays.

To define the individual positivity of each sample for the presence of SC, a cut-off (0.101) was defined based on the mean+2 SD median MFI values of the control group.

Supplementary Figure 1.

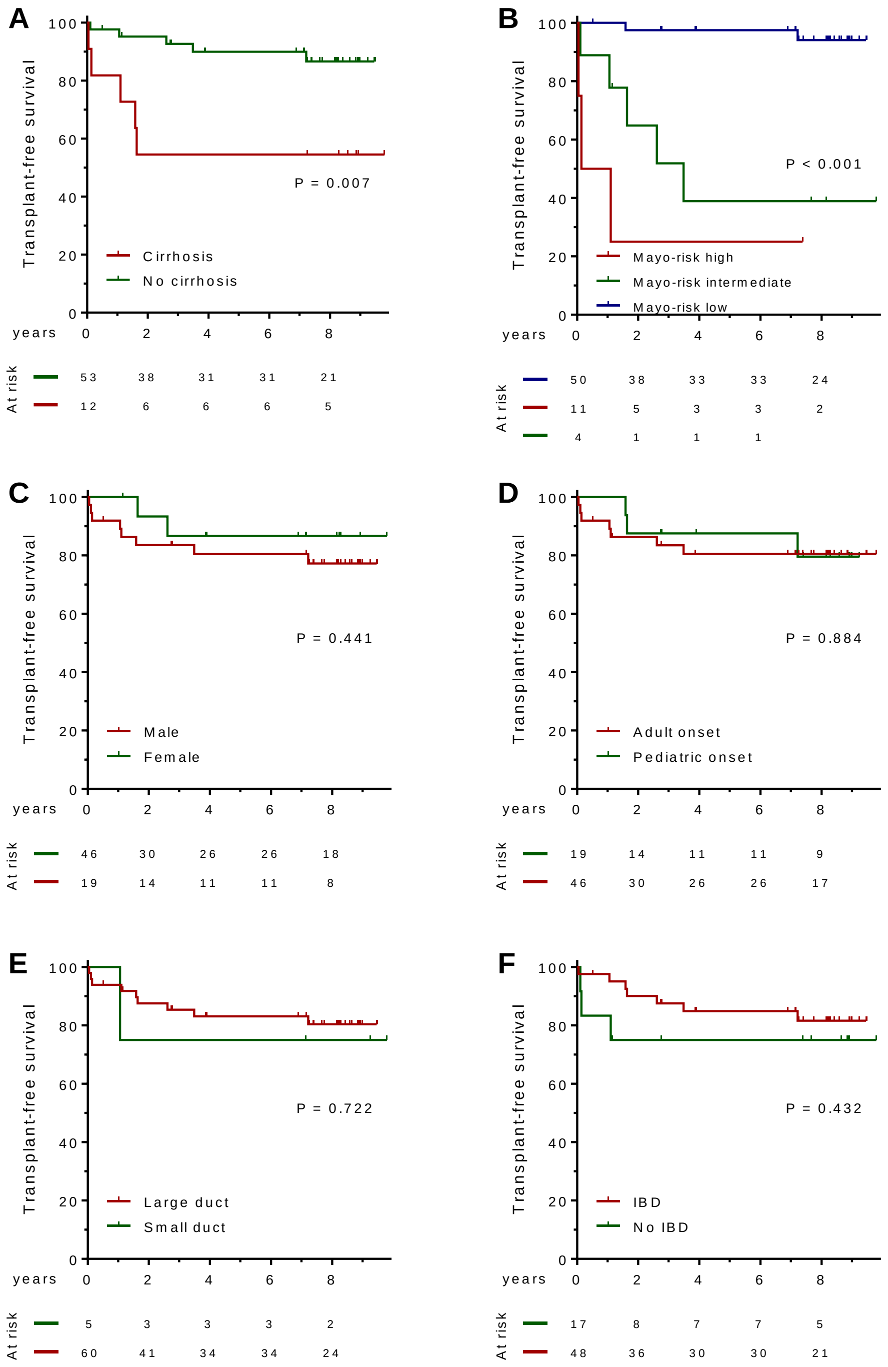

Supplementary Figure 1. Transplant free survival according to clinical variables. Mayo risk score and the presence of cirrhosis, but not gender, age at onset, disease location or concomitant IBD were significantly associated with faster disease progression. Cut-off for Mayo-score was: low risk: ≤0, intermediate risk: >0 and ≤2, high risk: >2. The cut-off for age at disease onset was 18 years, to differentiate pediatric onset and adult onset PSC.

Supplementary Table 1. Univariate Cox regression analysis of clinical variables in transplant-free survival of patients with PSC.

	Wald	HR	95% CI	P-value
Gender (female)	0.576	0.55	0.12 - 2.59	0.448
Age at onset (adult)	0.021	1.11	0.29 - 4.28	0.884
Presence of cirrhosis	5.954	4.7	1.36 - 16.29	0.015
Presence of IBD	0.603	0.59	0.15 - 2.27	0.437
Disease location (large duct)	0.125	0.69	0.09 - 5.44	0.723
Mayo-risk score	20.008	5.52	2.61 - 11.65	<0.001
Anti-CUZD1 IgA	0.899	1.93	0.5 – 7.47	0.343
Anti-CUZD1 IgG	0	1	0.21 – 4.73	0.998
Anti-GP2 IgA	5.614	5.15	1.33 – 19.97	0.018

References:

1. Komorowski, L. *et al.* Autoantibodies against exocrine pancreas in Crohn's disease are directed against two antigens: the glycoproteins CUZD1 and GP2. *J. Crohns. Colitis* **7,** 780–790 (2013).
2. Papp, M. *et al.* Evaluation of the combined application of ethanol-fixed and formaldehyde-fixed neutrophil substrates for identifying atypical perinuclear antineutrophil cytoplasmic antibodies in inflammatory bowel disease. *Clin. Vaccine Immunol.* **16,** 464–470 (2009).
